# Case Report: Ochronotic arthropathy mimicking spondyloarthritis: a case-based review of diagnostic pitfalls and a novel likely pathogenic HGD variant

**DOI:** 10.3389/fgene.2026.1762818

**Published:** 2026-05-07

**Authors:** Zhicheng Liu, Xinyu Xu, Yifan Yang, Sha Li, Ru Bai, Zhenghua Zhang, Yulong Guo, Ruomei Cui, Jian Xu, Shuang Liu

**Affiliations:** 1 Department of Rheumatology and Immunology, First Affiliated Hospital of Kunming Medical University, Kunming, Yunnan, China; 2 Medical Imaging Department, First Affiliated Hospital of Kunming Medical University, Kunming, Yunnan, China; 3 Department of cardiology, Fuwai Yunnan Hospital, Chinese Academy of Medical Sciences, Kunming, Yunnan, China

**Keywords:** alkaptonuria, homogentisate 1,2-dioxygenase, ochronosis, ochronotic arthropathy, osteoarthritis, spondyloarthropathies

## Abstract

**Background:**

Alkaptonuria (AKU) is a rare autosomal recessive metabolic disorder caused by homogentisate 1,2-dioxygenase (HGD) deficiency, leading to pigment deposition and progressive ochronotic arthropathy (OchA), which may mimic chronic inflammatory or degenerative joint diseases and delay diagnosis.

**Case description:**

A 48-year-old man with chronic low back pain and right knee arthritis was initially diagnosed with spondyloarthritis (SpA). Arthroscopy revealed black-brown synovial pigmentation initially overlooked. Subsequent clinical reassessment identified auricular cartilage pigmentation and progressive urine darkening, while imaging showed multilayered intervertebral disc calcification and degenerative changes, raising suspicion for AKU.

**Results:**

Genetic analysis detected a novel homozygous HGD missense variant (c.424A > G; p.Met142Val, exon 6), affecting a conserved residue and predicted to be deleterious. Classified as likely pathogenic according to ACMG criteria, it supported the diagnosis of OchA in conjunction with clinical, radiological and histopathological findings.

**Conclusion:**

This case highlights the diagnostic challenges of OchA mimicking SpA or osteoarthritis. Recognition of progressive urine darkening, cartilage pigmentation, and multilayered disc calcification may facilitate earlier diagnosis. The novel HGD variant expands the mutational spectrum of AKU and underscores the importance of molecular testing within a multidisciplinary framework in atypical joint degeneration. Although primarily metabolic, awareness of potential inflammatory involvement may guide symptomatic management when metabolic therapy is delayed.

## Highlights

- Ochronotic arthropathy masqueraded as seronegative spondyloarthritis; multimodal MDT evaluation was crucial for establishing the correct diagnosis of alkaptonuria.
- A novel homozygous HGD variant (c.424A > G; p.Met142Val), classified as likely pathogenic under ACMG criteria, was identified, potentially expanding the mutational spectrum and reinforcing the value of molecular testing in atypical cases.
- This case-based review highlights diagnostic pitfalls and discusses stage-specific inflammation-targeted management considerations when disease-specific therapy is unavailable.

## Introduction

Alkaptonuria (AKU), an extremely rare autosomal recessive metabolic disorder, has an estimated incidence of 1 in 250,000 to 1 in 1,000,000 globally. In mainland China, the condition is rarely reported, making clinical recognition even more challenging (Zatkova et al., 2012; Introne et al., 1993). AKU results from mutations in the homogentisate 1,2-dioxygenase (HGD) gene, a critical enzyme in tyrosine metabolism. HGD deficiency impairs the conversion of homogentisic acid (HGA) to maleylacetoacetic acid, leading to continuous HGA accumulation. HGA predominantly deposits in collagen-rich connective tissues, including soft tissues, cartilage, ligaments, tendons, and intervertebral discs, progressively inducing structural and functional degeneration (Bernardini et al., 2024). The classical clinical triad of AKU includes: (1) Homogentisic aciduria, characterized by excessive urinary HGA excretion, which oxidizes and polymerizes upon air exposure, turning urine dark brown to black; (2) Ochronosis, defined as bluish-black connective tissue pigmentation, most commonly observed in the auricles, sclerae, and skin; and (3) Ochronotic arthropathy (OchA), with HGA deposition in joint cartilage and synovium, causing progressive degenerative changes in the spine and major peripheral joints (Bernardini et al., 2024). Due to its mild and nonspecific clinical manifestations in childhood, AKU is often underdiagnosed. Progressive HGA accumulation leads to symptomatic joint involvement in adulthood, typically manifesting as pain and stiffness that gradually impact mobility and quality of life (Introne et al., 1993; Bernardini et al., 2024). Joint involvement predominantly affects large joints such as the spine, knees, and hips, with lumbar pain generally appearing around age 30 and knee pain after age 40 (Phornphutkul et al., 2002). Because early clinical manifestations are nonspecific, OchA is frequently misdiagnosed as osteoarthritis (OA) or spondyloarthritis (SpA). In many cases, diagnosis is not established until intraoperative observation of characteristic cartilage pigmentation, contributing to delayed management (Kostova et al., 2022).

We report a case of AKU that remained undiagnosed for over a decade, ultimately diagnosed through a multidisciplinary team (MDT) approach integrating imaging, histological examination, metabolic assessment, and genetic sequencing. We also conducted a comprehensive literature review to highlight the diagnostic challenges in differentiating OchA from OA and SpA, aiming to improve clinical recognition of rare metabolic arthropathies.

## Case presentation

**FIGURE 1:**
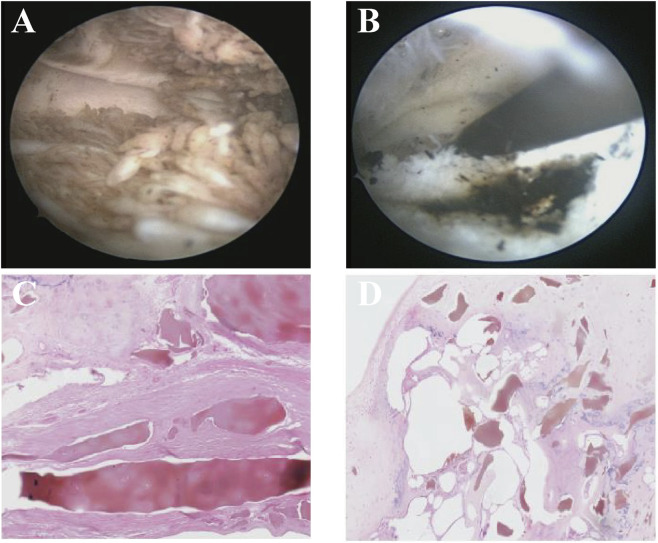
Arthroscopic and histopathological findings. **(A)** Articular cartilage showing yellow-brown pigmentation with surface irregularity. **(B)** Synovial membrane demonstrating diffuse black-brown pigment deposition. **(C,D)** Histopathology examination showing cartilage fragments containing yellow-brown pigment within synovial tissue, accompanied by fibrous hyperplasia, focal foreign body giant cell reaction, calcific deposits, ossification, osteophyte formation, architectural disruption, and partial chondroid metaplasia.

**FIGURE 2:**
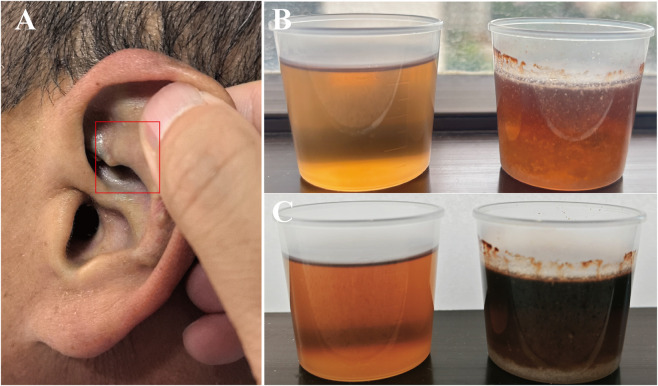
Clinical pigmentation and urine darkening test. **(A)** Bluish-black pigmentation of the left auricle cartilage. **(B)** Urine samples (30 mL each) after 4 h of air exposure: untreated sample (left) and sample alkalinized by addition of 2.5 g crushed sodium bicarbonate tablet (right). **(C)** Urine samples (30 mL each) after 24 h of air exposure: untreated sample (left) and alkalinized sample (right), demonstrating more rapid and pronounced darkening in the alkalinized specimen.

A 48-year-old manual laborer presented with intermittent dull lower back pain since 2009, worse at night and associated with morning stiffness and difficulty turning in bed. He did not undergo systematic medical evaluation initially and self-managed intermittently with non-steroidal anti-inflammatory drugs (NSAIDs), which provided only transient relief. From 2015, he developed bilateral knee pain, more pronounced on the right, activity-related and progressively worsening, initially suspected as OA. He sought care at multiple local hospitals, with gradually declining treatment efficacy. In October 2023, right knee arthroscopy was performed for MRI-detected loose bodies, ligament injuries, and meniscal tears. Intraoperatively, yellow-brown cartilage fragments and black-brown synovial pigment deposits were observed (Figures 1A,B), and histopathology confirmed pigmented cartilage (Figures 1C,D). These pathognomonic findings were initially overlooked, and no further diagnostic workup was conducted. The patient experienced no significant symptomatic relief post-surgery, prompting further consultations at multiple institutions. In July 2024, he was misdiagnosed with seronegative SpA and treated with adalimumab, etoricoxib, and sulfasalazine, without improvement. He was referred to our hospital in June 2025 for further evaluation. Family history was unremarkable, with no relatives exhibiting similar musculoskeletal or pigmentary features. Physical examination revealed bluish-black pigmentation of the left auricle (Figure 2A), restricted cervical and lumbar mobility (occiput-to-wall distance 17 cm), reduced right knee motion, and mild distal interphalangeal flexion deformities bilaterally. Two urine samples (30 mL each) were observed: one untreated and one alkalinized with crushed sodium bicarbonate (2.5 g added to achieve an alkaline environment). Both showed progressive darkening at 4 and 24 h, with more rapid and pronounced color change in the alkalinized sample (Figures 2B,C). No similar urine darkening was observed in any family members.

Laboratory tests showed white blood cell count (WBC) 9.72 × 10^9^/L (normal 3.5-9.5 × 10^9^/L), proteinuria (1+), urine microprotein 0.488 g/L (normal 0.028–0.065 g/L). Erythrocyte sedimentation rate (ESR) was 10 mm/h (normal 0–15 mm/h), C-reactive protein (CRP) 16.6 mg/L (normal ≤6 mg/L), and complement C3 1.5 g/L (normal 0.7–1.4 g/L). Autoimmune markers (HLA-B27, RF, ANA,ANCA, aPL, cytokine panel, TB-SPOT) were negative. Amino acid profiling revealed normal tyrosine and phenylalanine levels.

**FIGURE 3:**
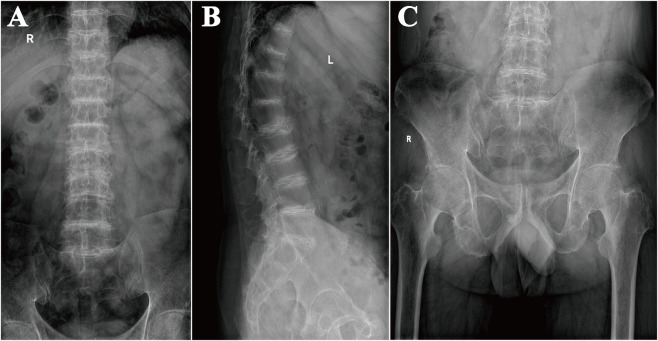
Radiographic findings of the lumbar spine and pelvis. **(A,B)** Lumbar spine radiographs demonstrating degenerative changes, including decreased bone density, loss of lumbar lordosis with sagittal imbalance, multilevel endplate sclerosis from T9 to S1, and intervertebral disc spaces narrowing with calcification. **(C)** Pelvic radiograph showing degenerative changes with symmetrical cortical irregularities and osteophyte formation at the bilateral iliac crests, greater trochanters, and ischial tuberosities.

**FIGURE 4:**
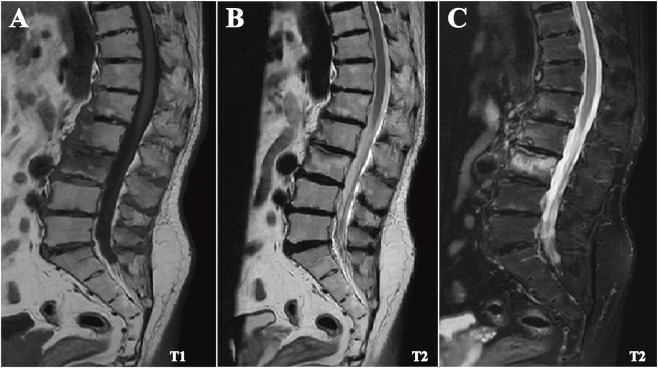
Lumbar spine MRI findings. **(A–C)** MRI of the lumbar spine demonstrating multilevel degenerative changes, including mild-to-moderate intervertebral disc space narrowing and reduced T2-weighted signal intensity of the discs, with thickening of the posterior longitudinal ligament. Mild anterior wedge deformities are observed at T11–L1, along with an L3 vertebral compression fracture. Fat-suppressed sequences show patchy hyperintensity within the L1-L3 vertebral bodies, consistent with bone marrow edema.

Imaging studies revealed multi-site degenerative changes. Lumbar X-ray demonstrated kyphosis, osteoporosis, narrowed disc spaces with calcifications, and marginal endplate sclerosis (Figures 3A,B), while pelvic X-ray revealed bilateral sacroiliac and hip joints degeneration (Figure 3C). Lumbar MRI demonstrated degenerative disc changes with reduced signal intensity, disc space narrowing, posterior longitudinal ligament thickening, bone marrow edema at L1-L3, and an L3 compression fracture (Figures 4A–C). Sacroiliac MRI confirmed bilateral degenerative changes. SPECT-CT showed increased uptake in shoulders, thoracolumbar and sacral vertebrae, knees, and feet, consistent with chronic inflammatory involvement. Knee ultrasound revealed osteophyte formation, mild synovial proliferation, low-grade synovitis, and minimal effusion. Chest CT and echocardiography were unremarkable.

Whole-exome sequencing revealed a homozygous missense variant, c.424A > G (p.Met142Val) in exon 6 of the HGD gene (NM_000187.4), affecting an evolutionarily conserved residue and predicted to be deleterious by multiple *in silico* tools (Table 1). According to American College of Medical Genetics and Genomics (ACMG) criteria (Richards et al., 2015), it is classified as likely pathogenic with the following evidence: PP3 (Strong), PM2 (Supporting), PM3 (Supporting). Functional validation and segregation analysis were not performed due to unavailable family samples. A heterozygous pathogenic ferrochelatase (FECH) variant c.362A > G (p.Glu121Gly), associated with erythropoietic protoporphyria (EPP), was detected incidentally.

**TABLE 1:** Summary of HGD gene variant identified by whole-exome sequencing.

Population frequency					
Databases	1000G	ESP6500	ExAC	GnomAD	GnomAD-EAS
Primary finding					
Homozygous missense variant in HGD (NM_000187.4:c.424A > G, p.Met142Val)					
Frequency value	-	-	-	-	-
Number of homozygous	-	-	-	-	-

In silico prediction								
Missense prediction			Cleavage site prediction			Nucleic acid conservative prediction		
SIFT	Mutation taster	Condel	SpliceAI	dbscSN_ RF	dbscSNV_ ADA	PhyloP vertebrates	PhyloP Placetal mammals	GERP++
deleterious	deleterious	deleterious	polymorphism	-	-	conserved	conserved	conserved

HGD, homogentisate 1,2-dioxygenase; 1000G, 1000 Genomes Project; ESP6500, Exome Sequencing Project; ExAC, exome aggregation consortium; GnomAD, genome aggregation database; GnomAD-EAS, GnomAD, East Asian. SIFT, sorting intolerant from tolerant; Condel, Consensus Deleteriousness; SpliceAI, splice artificial intelligence; dbscSNV_RF, database of splicing consensus SNVs–Random Forest; dbscSNV_ADA, database of splicing consensus SNVs–AdaBoost; PhyloP, Phylogenetic P-values; GERP, genomic evolutionary rate profiling; -, indicates absence or zero.

Together with the clinical triad, arthroscopic and pathological pigmentation, and imaging findings, these genetic results support the diagnosis of AKU presenting as OchA.

### Search strategy

To identify diagnostic pitfalls of OchA misdiagnosed as SpA or OA, a systematic literature search was conducted in the PubMed and Web of Science databases for publications from January 2005 to July 2025, with emphasis on the past decade. Search terms included: alkaptonuria; ochronosis; ochronotic arthropathy; spondyloarthropathy; ankylosing spondylitis; and osteoarthritis.

Eligibility criteria were: (1) case reports or case-based reviews on OchA; (2) explicit discussion of its differential diagnosis from other chronic joint disorders; (3) English-language articles; and (4) full-text availability. Selected studies were analyzed for patient characteristics, diagnostic approaches, imaging and histopathological features, and genetic findings to summarize key clinical and diagnostic insights.

## Discussion

AKU was first described by Rudolf Virchow in 1866 (Virchow, 1966), and later recognized as a Mendelian metabolic disorder by Archibald Garrod in 1902 (Garrod, 1902). OchA, a progressive joint manifestation of AKU, arises from HGA deposition in cartilage and connective tissues. This accumulation reduces tissue elasticity and biomechanical integrity, induces chronic synovitis and drives progressive degeneration of cartilage, ligaments, and tendons, ultimately resulting in joint destruction and functional impairment (Ranganath et al., 2019; Salem and Elmoghazy, 2025; Davison and Norman, 2023; Ventura-Ríos et al., 2016; Jiang et al., 2019; Balaban et al., 2006).

In its early stages, OchA predominantly affects the spine, with chronic low back pain as the most common presenting symptom. Progressive loss of lumbar lordosis, kyphosis, reduced mobility, and height loss may follow (Yuce et al., 2021; Chu et al., 2021). In the present case, the patient developed chronic low back pain in his early thirties with gradual worsening and decreasing response to conventional analgesic and anti-inflammatory therapies, consistent with the typical spinal course of AKU (Phornphutkul et al., 2002). Notably, darkened urine and subtle bluish-black auricular cartilage pigmentation were only recognized during the current evaluation, highlighting how early non-specific manifestations may be overlooked. Ochronotic spondyloarthropathy has been described as progressing through four stages——an inflammatory phase, early disc calcification, fibrous ankylosis, and eventual osseous ankylosis (Tanios et al., 2023). In advanced disease, the radiographic manifestations may closely mimic those observed in ankylosing spondylitis (AS), increasing the likelihood of diagnostic misclassification (Tanios et al., 2023). Characteristic radiographic findings of OchA include multilevel intervertebral disc calcifications and the vacuum phenomenon, often accompanied by progressive degenerative changes (Salem and Elmoghazy, 2025; Chu et al., 2021). In the present case, lumbar imaging demonstrated multistage disc calcifications, while MRI revealed patchy hyperintensity at L1-L3 corresponding to bone marrow edema and an L3 compression fracture. Although these findings were initially interpreted as chronic degenerative changes, they likely represented early manifestations of OchA. Their diagnostic significance was underestimated and not further investigated, contributing to delayed recognition. As the disease progresses, additional structural changes—including disc space narrowing or fusion, facet joint degeneration, and osteophyte formation—become increasingly apparent (Borman et al., 2002). When lumbar involvement predominates, these features may closely resemble seronegative SpA (Ventura-Ríos et al., 2016; Balaban et al., 2006; Chu et al., 2021). Despite overlapping radiographic patterns, several distinctions remain clinically meaningful. AS is characterized by early sacroiliac joints (SIJs) erosions and joint space narrowing followed by progressive fusion, vertebral squaring, marginal inflammation, and vertically oriented syndesmophytes that may culminate in a bamboo spine appearance (Balaban et al., 2006; Tanios et al., 2023; Borman et al., 2002). Although SIJs were traditionally considered spared in OchA and thus used as a key differentiating feature (Salem and Elmoghazy, 2025; Yuce et al., 2021), subsequent reports have demonstrated that mild SIJ space narrowing and subchondral sclerosis may also occur in OchA (Yuce et al., 2021; Damian et al., 2013; İnel et al., 2021). Thus, reliance on SIJ involvement alone is insufficient for differential diagnosis; attention to erosive changes and vertically oriented syndesmophytes is more informative. When multisegmental disc calcifications occur with SpA-like features but without systemic inflammation or immunologic abnormalities, a metabolic disorder such as AKU should be considered. Early recognition of these features is essential to avoid diagnostic delay, as illustrated in this case.

As AKU progresses, OchA commonly affects large- and medium-sized peripheral joints, most frequently the knees, presenting with pain, stiffness, and occasional synovial effusion (Chu et al., 2021). In this patient, progressive right knee pain with limited mobility developed in his forties, consistent with the typical peripheral involvement of OchA (Phornphutkul et al., 2002). OA remains the primary differential diagnosis due to clinical and radiographic overlap, including chronic pain in the spine, knees, and hips, as well as joint space narrowing and subchondral sclerosis (Salem and Elmoghazy, 2025; Ventura-Ríos et al., 2016). Key distinctions include age of onset—OA usually occurs after 60, whereas OchA manifests earlier (30–50 years)—and etiology, with OchA driven by metabolic pigment rather than mechanical stress (Kostova et al., 2022; Chu et al., 2021). In this case, arthroscopy and histology revealed black-brown synovial pigment and yellow-brown cartilage fragments; their significance was initially overlooked, likely due to the rarity of AKU and limited clinical suspicion among treating clinicians. This diagnostic oversight illustrates how cognitive bias and gaps in awareness of rare metabolic arthropathies can delay recognition. Radiographically, spinal OA typically demonstrates slowly progressive segmental disc degeneration, whereas disc calcification and vacuum phenomenon are uncommon (Chu et al., 2021).

To date, over 250 pathogenic or likely pathogenic HGD mutations have been reported, predominantly missense variants, summarized in the HGD mutation database (http://hgddatabase.cvtisr.sk/) (Zatkova et al., 2012). HGD mutations are rarely reported in Asia, particularly in the Chinese population (Yang et al., 2013). High-throughput sequencing has increasingly facilitated molecular confirmation of AKU. In this patient, a novel homozygous missense variant c.424A > G (p.Met142Val) in exon 6, affecting an evolutionarily conserved residue and predicted deleterious by multiple *in silico* tools, was identified. Structural analysis suggests that this substitution may disrupt HGD enzyme stability or the catalytic site, supporting the genotype-phenotype correlation. Its rarity, homozygous status, and concordance with clinical, imaging, and histopathological findings support diagnosis of AKU and highlight the value of molecular testing in rare arthropathies. Functional validation was not performed, and family testing was unavailable, leaving the inheritance pattern unconfirmed (Gómez-Lechón Quirós et al., 2021). Additionally, a heterozygous pathogenic FECH variant associated with EPP was detected, which may warrant family evaluation despite the absence of phenotype.

The patient’s diagnosis spanned over a decade, highlighting the challenges of recognizing rare metabolic arthropathies. He was initially misdiagnosed with seronegative SpA, despite negative HLA-B27 results and imaging findings that were inconsistent with SpA, underscoring the phenotypic overlap between early-stage OchA and common inflammatory rheumatic diseases. Clinicians should maintain vigilance when encountering: (i) degenerative joint disease involving multiple sites in young to middle-aged adults (typically 30–50 years) with slow progression; (ii) atypical imaging features that deviate from patterns seen in OA or SpA; and (iii) pigment deposition in cartilage detected *via* arthroscopy or histopathology, especially when conventional therapies are ineffective. In this case, the lack of a coordinated multidisciplinary approach—encompassing rheumatology, orthopedics, radiology, pathology, and medical genetics—contributed to diagnostic delay. This underscores the essential role of MDT framework in rare metabolic disorders, which integrates clinical assessment, imaging interpretation, biochemical and metabolic evaluation, and genetic testing, thereby enhancing diagnostic accuracy and enabling earlier recognition.

No definitive cure currently exists for AKU and management primarily focuses on symptom control and slowing disease progression, with total joint replacement reserved for advanced OchA (Salem and Elmoghazy, 2025; Keller et al., 2005). In this case, long-term treatment with NSAIDs, tumor necrosis factor-alpha (TNF-α) inhibitors (adalimumab), and sulfasalazine provided only transient or diminishing relief. Although OchA has traditionally been regarded as a non-inflammatory disorder, pigment-induced structural degeneration may coexist with mild HGA-triggered inflammatory responses (Yuce et al., 2021). Accordingly, a short-term empirical trial of an interleukin-17 (IL-17) inhibitor (Ixekizumab) was undertaken after thorough counseling, resulting in transient symptom improvement. This transient benefit likely reflects modulation of low-grade HGA-induced inflammation rather than disease modification, suggesting that targeting inflammatory pathways may confer stage-specific benefit in selected patients, although its indication and efficacy in OchA remain uncertain, and symptomatic relief may obscure the underlying metabolic pathology. Targeted metabolic therapy with nitisinone (NTBC), a 4-hydroxyphenylpyruvate dioxygenase (HPPD) inhibitor, has demonstrated robust reductions in serum and urinary HGA levels in SONIA 1 and SONIA 2 trials, mitigating progressive ochronotic tissue damage (Sloboda et al., 2019; Introne et al., 2011; Ranganath et al., 2020; Ranganath et al., 2016). NTBC has been approved for the treatment of adult AKU patients in Europe and North America (Ranganath et al., 2020; Ranganath et al., 2016); although it has been approved for marketing in mainland China, it currently lacks an indication for AKU, which may limit its routine clinical use for this condition. While its biochemical efficacy is well documented, the extent to which long-term clinical outcomes are modified requires further longitudinal evaluation (Keller et al., 2005; Mayrink et al., 2025). In this patient, subsequent follow-up after initiation of NTBC therapy resulted in substantial symptom improvement, illustrating the potential clinical benefit of upstream metabolic intervention. Emerging strategies, including gene-editing, enzyme replacement, and RNA-based therapies, remain under investigation and may represent future etiological treatment directions in AKU (Bernardini et al., 2024; Martinez-Pizarro and Desviat, 2022).

While the clinical and genetic findings strongly support the diagnosis, the absence of functional validation of HGD activity in liver or kidney cells, as well as family-based segregation data, limits the definitive pathogenic classification of the p.Met142Val variant, which is therefore considered “likely pathogenic” according to ACMG criteria. Addressing these limitations in future studies through functional assays and family analyses would further strengthen the molecular evidence and clarify causal relationships.

## Conclusion

This case highlights that OchA, a manifestation of AKU, can present with nonspecific degenerative changes mimicking SpA or OA, often leading to delayed diagnosis. Key early indicators, including progressive urine darkening, bluish-black cartilage pigmentation, and multilayered intervertebral disc calcification, should prompt consideration of metabolic arthropathies. Molecular diagnostics combined with an MDT approach were critical for accurate diagnosis and may help inform clinical practice for timely recognition of rare metabolic arthropathies aiding differentiation from more common degenerative and inflammatory conditions.

## Data Availability

The original contributions presented in the study are included in the article/supplementary material, further inquiries can be directed to the corresponding authors.
